# Violence and Mental Health Among Gender-Diverse Individuals Enrolled in a Human Immunodeficiency Virus Program in Karnataka, South India

**DOI:** 10.1089/trgh.2018.0051

**Published:** 2019-11-06

**Authors:** Laura H. Thompson, Sumit Dutta, Parinita Bhattacharjee, Stella Leung, Anindita Bhowmik, Ravi Prakash, Shajy Isac, Robert R. Lorway

**Affiliations:** ^1^ Department of Community Health Sciences, Centre for Global Public Health, University of Manitoba, Winnipeg, Canada.; ^2^ Department of Social Studies, Dr. K.N. Modi University, Newai, India.; ^3^ Philips Innovation Campus, Bangalore, India.; ^4^ Karnataka Health Promotion Trust, Bangalore, India.

**Keywords:** hijra, India, kothi, mental health, transgender, violence

## Abstract

**Purpose:** Gender-diverse individuals in India face considerable discrimination, stigma, and violence. There is a dearth of published literature describing experiences of violence among this population and potential links to mental health.

**Methods:** A questionnaire was administered to 282 study participants, 18 years of age and older, who self-identified as hijra, kothi, double decker, or bisexual and were actively enrolled in a local HIV prevention program in Bangalore, India in 2012. Responses were used to calculate a composite depression/anxiety score. Associations between sociodemographic characteristics and experiences of physical and sexual violence in the previous six months were tested and differences in depression/anxiety score based on experiences of violence were explored.

**Results:** Recent physical violence was common among study participants and was reported among 46% of nirvan (emasculated) hijras (transgender), 42% of akwa (not emasculated) hijras, and 25% of kothis (feminine acting males). Rape in the previous year was particularly common among akwa hijras (39%). Factors associated with being raped included younger age, less education, and employment in basti (blessings), sex work, chela (disciple of hijra guru), or at a community-based organization. Kothis had the highest depression/anxiety score. No significant difference in depression/anxiety score based on recent history of physical violence or rape was found.

**Conclusions:** Physical violence and poor mental health are common among gender-diverse individuals in Bangalore, Karnataka. There is a need for services that cater to the unique mental health needs of gender-diverse individuals in India, following rights-based approaches that address the underlying roots of oppression they encounter.

## Introduction

India is home to gender-diverse males and transgender individuals with a range of identities unique to South Asia, including hijras and kothis, who are highly marginalized in Indian society.^1–3^ In 2011, it was estimated that 488,000 of the 1.25 billion residents of India were transgender,^4^ although likely an underestimate.

Hijras, who have been described by some scholars as a “third gender”,^5^ are usually born as males or intersex and may identify as transgender, women, “in-between men and women,” or “neither man nor woman.”^6^ Hijras publicly wear female attire, behave in a feminine way, and may engage in distinctive behaviors such as clapping when seeking attention, begging at traffic signals and railway stops, and performing “basti” by blessing shops and other commercial establishments in exchange for money. Some hijras, termed nirvan hijras, voluntarily undergo the ritual of emasculation involving the excision of the penis and testicles, whereas others (akwa hijras) have not undergone emasculation. Senior members of the hijra community may be referred to as “gurus” and may have one or more followers or disciples. These disciples, referred to as “chelas”, may claim either a hijra or a kothi identity and serve the guru, providing a share of their earnings.^6^ Anyone who wishes to join the hijra community is required to choose a senior member of the community to act as their guru.

Kothis are a heterogeneous group of feminine males with same-sex attraction who, unlike hijras, usually wear male clothing in public, although they may cross-dress at private community gatherings or while practicing sex work and may identify as transgender.^7^ Kothis may be married to a woman.^8^

Despite the fact that gender-diverse identities have existed in India for thousands of years and are not forbidden in Hinduism, Indian society generally is intolerant toward those who do not conform to heteronormative identities^2,5^ and this stigmatizes and marginalizes hijra and kothi identities. Although some marry women for social and economic reasons, hijras and kothis typically prefer male sexual partners.

Hijras and kothis often project a feminine persona in their everyday lives, not only making them visible to potential sexual partners but also increasing their vulnerability to violence, discrimination, and emotional and physical abuse. Facing abuse or disownment by their immediate families, hijras and kothis often leave their homes, missing out on educational opportunities and entering poverty and homelessness and increasing marginalization.^1,9,10^ Many perform sex work for their livelihoods, which may introduce additional layers of stigmatization and risk of violence.^11,12^ Discrimination in public and private health care and social service settings is common, reducing access to essential services and perpetuating the effects of internalized stigma.^11,13^ Experiences of violence are common among gender-diverse individuals in India.^14^ The perpetrators of violence include family members, members of the public, gurus, sexual and intimate partners (“panthis”^*^), and police.^1^ Along with inflicting physical and sexual violence, police, sexual partners, and sometimes gurus extort money through blackmailing and other forms of exploitation.^1,11^

Generally, gender-diverse individuals have been found to be at a greater risk of mental health issues due to longstanding exposure to stigma, discrimination, violence, and psychological distress.^15–20^ A lack of social support from within communities of sexual minority groups in India has been reported and may further exacerbate the effects of stigma, discrimination, and violence on mental health.^11,16,17^ Previous studies have reported that a lack of social support, economic disadvantage, and transphobia are largely interconnected and correspond to depression and suicidal behavior.^21–23^

Meyer's Minority Stress Theory describes how members of stigmatized minority groups are subjected to chronic psychosocial stress in the context of pervasive prejudice, discrimination, and stigma within their social environments.^24^ There is evidence that while stress exposure is related to experiences of prejudice, different subgroups—such as ethnic minorities—may be disproportionately affected, and the resources that individuals have to cope with stress are often socially patterned.^25^ By identifying with and participating in their communities, members of stigmatized minority groups may access group-level coping resources such as social spaces with affirmative values and norms and the validation and reappraisal of stressful experiences and feelings.^26^ Indeed, a study conducted among men who have sex with men (MSM) in two sites in Tamil Nadu, South India, revealed that participants in the urban area of Chennai, which was less conservative and had more services for MSM than the semiurban area of Kumbakonam, reported significantly higher levels of social support and resilient coping, and these were associated with lower depression.^27^ Another study conducted among trans women and MSM in urban and rural sites in India found an association between sexual and gender minority stigmas and depression, mediated by social support and resilient coping.^28^

In 2005, the National Commission on Macroeconomics and Health reported that there were 60 million people in India with a mental illness,^29^ and it is estimated that between 70% and 90% do not receive treatment.^30–32^ More recent data, collected in 2015/2016 as part of the National Mental Health Survey of India, indicate that the burden of mental illness remains high in India. Among the 12 states of India that were included in the survey, the average lifetime morbidity of mental illness was 14% and ranged from 8% in Assam to 20% in Manipur.^31,32^ Despite the large burden of mental illness in India, mental health care has been underresourced, composing only 0.06% of the total health budget in 2011.^33^ Due to a lack of prioritization, mental health care is limited in primary health care and community settings, with poor funding, a lack of skilled human resources and leadership, and poor access to mental health services.^34–36^

The signing of the United Nations Convention on the Rights of Persons with Disabilities by India in 2007 led to the establishment of a mental health policy and action plan in 2014^37,38^ and the Mental Healthcare Act, which passed unanimously on March 30, 2017.^37^ The mental health policy calls for effective governance and accountability, promotion of mental health, prevention of mental disorders and suicide, universal access to mental health services, enhanced availability of human resources for mental health, and community participation and has identified marginalized groups as those who are economically poor or homeless.^38^ The Mental Healthcare Act establishes a right to access mental health care regardless of “gender, sex, sexual orientation, religion, culture, caste, social or political beliefs, class, disability, or any other basis,” compelling state governments to provide services by January 7, 2018.^37,39^ This is a promising first step, and action is required to ensure accountability to the Mental Healthcare Act, and genuinely equitable access to mental health care, particularly for highly stigmatized individuals.

This article describes the mental health and experiences of violence of a sample of gender-diverse individuals in Bangalore, South India. Potential links between mental health and experiences of violence are also explored. This evidence may be used to inform the design of mental health services tailored to the specific needs of gender-diverse individuals in South India.

## Methods

### Study design and sampling

A cross-sectional study was conducted using a structured questionnaire to collect information about experiences of violence and mental health among gender-diverse individuals in Bangalore, Karnataka. Any individual who was 18 years of age and older and who was enrolled and active in the HIV prevention program implemented for gender-diverse individuals by the Karnataka Health Promotion Trust at the time of the study (2012) was eligible to participate in the study. Stratified simple random sampling was used to select prospective participants after stratifying them by gender subtype (nirvan hijra, akwa hijra, kothi, and “other,” which included individuals who were enrolled in the program, in the same networks as the hijra and kothi participants, and who chose to self-identify as double decker or bisexual).

Seven hijra and kothi community members were recruited as interviewers based on their knowledge of the local language Kannada, rapport within the community, sensitivity, and knowledge of research ethics and norms of confidentiality. They were provided 6 days of intensive training to prepare them for the data collection process that adhered to the ethical principles of conducting research on a sensitive subject.

To protect the privacy of study participants, the questionnaires were administered after obtaining informed written consent at one of the following locations: the drop-in center of the targeted intervention program, a hamam, or the home of the study participant. The hijra participants tended to choose a hamam or their home as the location for the interview, whereas kothi participants, many of whom live with their families, preferred the drop-in center. A total of 282 individuals were interviewed individually in English or the local language, Kannada, by trained peer workers using a structured questionnaire. Each participant received an honorarium of 300INR (∼$4.30 USD), to compensate them for their time and any travel-related expenses incurred. The Institutional Ethical Review Board of St. John's Medical College in Bangalore, India, approved this study on April 12, 2012 (reference # 48/2012).

### Measures

The questionnaire collected demographic characteristics, perception of body image, self-esteem, relationships, alcohol consumption, health, anxiety and depression, suicidality, violence, psychosocial coping strategies, and exposure to HIV intervention programs. Demographic variables included gender identity, age, religion, education level, main income source, history of being married to a woman, and living arrangement. Study participants were asked how many times they had experienced physical violence in the previous 6 months and how many times they had been raped in the previous 12 months. Due to the distribution of responses, these violence and rape variables were converted into binary variables capturing whether or not they had been experienced by the participant.

The questions that were focused on aspects related to depression and anxiety were derived from the World Health Organization's Quality of Life Scale (WHOQOL-HIV),^40^ the Hamilton Anxiety Scale (HAM-A),^41^ and the Beck Depression Inventory (BDI-II).^42^ These scales have not been adapted to the Indian context. A formative qualitative contextualization process based on prior focus group discussions and in-depth interviews with community members was used to select a set of questions from the three standard scales, grounding the questionnaire in the current sociopolitical realities within the community. As a result, no single complete standard scale appears in the questionnaire. Factor analysis was conducted on this series of questions to identify a set of questions that could be used to calculate a composite mental health score measuring symptoms of depression and anxiety for participants. The Cronbach's alpha for the resulting scoring system was 0.7317. A composite depression and anxiety score was calculated by adding together the number of selected questions that the participant indicated a “yes” response for, with a maximum possible value of 18. The depression and anxiety score is not a diagnosis and instead provides a means of measuring the degree, to which study participants experienced depressive symptoms and feelings of anxiety.

### Data analysis

Descriptive analyses were conducted using STATA version 11. Fisher's Exact tests were used for bivariate tests of associations between sociodemographic characteristics and experience of physical violence in the previous 6 months and rape in the previous 12 months. The frequencies of responses to every question that was included in the depression and anxiety score were calculated for the overall sample and by gender identity and are presented in a table. Two-way analysis of covariance (ANCOVA) was used to determine whether there were statistically significant differences in mean depression and anxiety score based on experiences of violence for each gender identity category, adjusting for the two variables that were significantly associated with experiences of violence in the Fisher's Exact tests: age and income source.

## Results

### Characteristics of study participants

Of the 282 participants, 38.3% self-identified as hijra (27.3% nirvan and 11.0% akwa) and 48.6% self-identified as kothi (Table 1). Thirteen percent grouped into the “other” category, and commonly self-identified as “double decker”^†^ or bisexual. Nirvan hijras and kothis tended to be younger, with 59.7% and 52.6%, respectively, younger than the age of 30.

**Table 1.** Characteristics of Study Participants

Characteristic	Nirvan hijra *N*=77 (27.3%)	Akwa hijra *N*=31 (11.0%)	Kothis *N*=137 (48.6%)	Other identities *N*=37 (13.1%)	All participants *N*=282
Age (years)					
<30	46 (59.7)	13 (41.9)	72 (52.6)	16 (42.2)	147 (52.1)
30+	31 (40.3)	18 (58.1)	65 (47.4)	21 (56.8)	135 (47.9)
Mean age (SD)	32.8 (31.1–34.5)	30.8 (28.3–33.4)	31.5 (30.0–33.0)	30.2 (27.9–32.6)	31.6 (30.7–32.5)
Religion					
Hindu	66 (85.7)	27 (87.1)	118 (86.1)	30 (81.1)	241 (85.5)
Muslim	5 (6.5)	3 (9.7)	12 (8.8)	6 (16.2)	26 (9.2)
Christian	6 (7.8)	1 (3.2)	5 (3.7)	6 (16.2)	13 (4.6)
Education (*n*=281)					
None/illiterate	15 (19.7)	2 (6.5)	27 (19.7)	3 (8.1)	47 (16.7)
1–8 Years	20 (26.3)	9 (29.0)	23 (16.8)	11 (29.7)	63 (22.4)
9–10 Years	23 (30.3)	7 (22.6)	27 (19.7)	13 (35.1)	70 (24.9)
11+ Years	18 (23.7)	13 (41.9)	60 (43.8)	10 (27.0)	101 (35.9)
Main income source					
None/student	0 (0)	0 (0)	12 (8.8)	1 (2.7)	13 (4.6)
Laborer/transport	1 (1.3)	1 (3.2)	20 (14.6)	1 (2.7)	23 (8.2)
Service/professional/trade	0 (0)	1 (3.2)	94 (68.6)	30 (81.1)	125 (44.3)
Basti/sex work/chelas/CBO	76 (98.7)	29 (93.6)	11 (8.0)	5 (13.5)	121 (42.9)
Ever married to a woman					
Yes	73 (94.8)	25 (80.7)	85 (62.0)	26 (70.3)	209 (74.1)
No	4 (5.2)	6 (19.3)	52 (38.0)	11 (29.7)	73 (25.9)
Living arrangement (*n*=281)					
Living alone	16 (20.8)	12 (38.7)	24 (17.5)	2 (5.4)	54 (19.2)
Living with family	1 (1.3)	4 (12.9)	72 (52.6)	20 (54.1)	97 (34.4)
Living with female spouse	0 (0)	1 (3.2)	31 (22.6)	8 (21.6)	40 (14.2)
Living with guru	27 (35.1)	4 (12.9)	0 (0)	2 (5.4)	33 (11.7)
Living with a male partner	8 (10.4)	3 (9.7)	1 (0.7)	1 (2.7)	13 (4.6)
Living with friends	15 (19.5)	7 (22.6)	9 (6.6)	4 (10.8)	35 (12.4)
Living with chelas	9 (11.7)	0 (0)	0 (0)	0 (0)	9 (3.1)

CBO, community-based organization; SD, standard deviation.

More than 40% of kothis and akwa hijras had achieved the highest levels of education, although one-fifth of kothis and nirvan hijras had no education. The nirvan (98.7%) and akwa (93.6%) hijras were mostly employed in basti, chelas, sex work, or in a community-based organization (CBO) setting. However, those identifying as kothi (68.6%) and other (double decker or bisexual) (81.1%) mostly worked in the service industry or business. The most common living arrangement for nirvan hijras (35.1%) was with a guru, whereas akwa hijras (38.7%) tended to live alone. More than half of all kothis (52.6%) and those in the “other” (double decker or bisexual) category (54.1%) reported living with family, and 22% lived with a female spouse.

### Sociodemographic factors associated with experiences of violence

Physical violence in the previous 6 months was extremely common among participants identifying as nirvan hijra (46.1%) and akwa hijra (41.9%) (Table 2). A quarter of all kothis had experienced physical violence in the previous 6 months and nearly 9% of participants in the “other” (double decker or bisexual) identity category reported experiencing physical violence in the previous 6 months. Out of all the respondents, the largest proportion of those who had experienced rape in the previous year identified as akwa hijras (38.7%), while ∼20% of participants in all of the other identity categories had.

**Table 2.** Bivariate Associations Between Sociodemographic Characteristics and Experiences of Violence Using Fisher's Exact Tests

Characteristic	Physical violence,^a^ 6 months, *N*=83 Count, expected count (row %)	*p*-Value	Raped, 12 months, *N*=62 Count, expected count (row %)	*p*-Value
Identity		*p*=0.000		*p*=0.139
Nirvan	35, 23 (46.1)		17, 16 (23.6)	
Akwa	13, 9 (41.9)		12, 7 (38.7)	
Kothi	32, 41 (23.5)		25, 30 (18.8)	
Other	3, 10 (8.6)		8, 8 (22.2)	
Age group (years)		*p*=0.050		*p*=0.060
<30	47, 39 (35.6)		37, 30 (28.0)	
30+	36, 44 (24.7)		25, 32 (17.9)	
Education (*n*=281)		*p*=0.114		*p*=0.070
None/illiterate	9, 14 (19.6)		9, 10 (20.5)	
1–8 Years	20, 18 (32.3)		19, 14 (32.2)	
9–10 Years	27, 20 (39.1)		19, 16 (27.1)	
11+ Years	26, 30 (26.0)		15, 22 (15.3)	
Main income source		*p*=0.000		*p*=0.012
None/student	4, 4 (30.8)		1, 3 (8.3)	
Laborer/transport	10, 7 (43.5)		5, 5 (22.7)	
Service/professional/trade	18, 36 (14.8)		19, 28 (15.5)	
Basti/sex work/chelas/CBO	51, 36 (42.5)		37, 26 (32.2)	

^a^ Physical violence was defined in the questionnaire as hurt, hit, slapped, pushed, kicked, punched, choked, burned—but not with a weapon.

Identity, age group, and income source were associated with having experienced physical violence in the previous 6 months (Table 2). Physical violence was experienced in the previous 6 months significantly more among younger people younger than 30 years of age and among nirvan and akwa hijras compared to kothis and “other” (double decker or bisexual) identities. Physical violence was experienced in the previous 6 months significantly less among people with employment in the service industry, professionals, and those in trades.

Factors associated with being raped in the previous 12 months included younger age and employment in basti, sex work, chela, or with a CBO.

### Mental health issues among study participants

Generally, study participants who selected “other” for the gender identity question (i.e., identify as double decker or bisexual) more frequently reported never having experienced these mental health issues compared to hijra and kothi participants (Table 3). Nirvan hijras and kothis tended to follow the same patterns for some mental health issues, with the greatest proportions reporting “constantly” worrying about things (46.1% and 51.8%, respectively), fatigue/lack of energy (19.5% and 17.7%, respectively), lack of self-confidence (16.9% and 16.2%), and trouble sleeping (20.8% and 16.8%). Among hijras and kothis, 23–32% reported “always” anticipating the worst, 17–24% reported feeling that life is not worth living, and 19–26% reported a lack of concentration. When asked, more than 40% of kothis reported always feeling saddened while 29–33% of nirvan and akwa hijras, respectively, reported the same. A loss of interest in daily activities was most commonly reported among kothis (18.3%).

**Table 3.** Mental Health of Study Participants

Mental health condition	Frequency	Nirvan hijra (%) *N*=77	Akwa hijra (%) *N*=31	Kothis (%) *N*=137	Other identities (%) *N*=37	All (%) *N*=282
Constantly worry about things	Never	19 (25.0)	4 (12.9)	12 (8.8)	11 (29.7)	46 (16.4)
	Sometimes	22 (29.0)	16 (51.6)	54 (39.4)	24 (64.9)	116 (41.3)
	Always	35 (46.1)	11 (35.5)	71 (51.8)	2 (5.4)	119 (42.4)
Anticipating the worst in any situation	Never	22 (28.6)	10 (32.3)	32 (23.4)	13 (35.1)	77 (27.3)
	Sometimes	33 (42.9)	11 (35.5)	73 (53.3)	21 (56.8)	138 (48.9)
	Always	22 (28.6)	10 (32.3)	32 (23.4)	3 (8.1)	67 (23.8)
Saddened	Never	16 (21.1)	5 (16.7)	10 (7.4)	4 (10.8)	35 (12.6)
	Sometimes	38 (50.0)	15 (50.0)	68 (50.4)	29 (78.4)	150 (54.0)
	Always	22 (29.0)	10 (33.3)	57 (42.2)	4 (10.8)	93 (33.5)
Loss of interest in daily activities	Never	34 (44.2)	16 (51.6)	47 (34.3)	25 (69.4)	122 (43.4)
	Sometimes	33 (42.9)	13 (42.0)	65 (47.5)	7 (19.4)	118 (42.0)
	Always	10 (13.0)	2 (6.5)	25 (18.3)	4 (11.1)	41 (14.6)
Fatigue/lack of energy	Never	32 (41.6)	19 (61.3)	54 (39.7)	25 (67.6)	130 (46.3)
	Sometimes	30 (39.0)	10 (32.3)	58 (42.7)	10 (27.0)	108 (38.4)
	Always	15 (19.5)	2 (6.5)	24 (17.7)	2 (5.4)	43 (15.3)
Lack of self-confidence	Never	41 (53.3)	22 (71.0)	62 (45.6)	22 (61.1)	147 (52.5)
	Sometimes	23 (29.9)	8 (25.8)	52 (38.2)	13 (36.1)	96 (34.3)
	Always	13 (16.9)	1 (3.2)	22 (16.2)	1 (2.8)	37 (13.2)
Feel life is not worth living	Never	32 (41.6)	7 (23.3)	49 (35.8)	10 (27.0)	98 (34.9)
	Sometimes	27 (35.1)	18 (60.0)	61 (44.5)	25 (67.6)	131 (46.6)
	Always	18 (23.4)	5 (16.7)	27 (19.7)	2 (5.4)	52 (18.5)
Lack of concentration	Never	18 (23.7)	10 (32.3)	19 (14.0)	10 (27.0)	57 (20.4)
	Sometimes	40 (52.6)	13 (41.9)	91 (66.9)	23 (62.2)	167 (59.6)
	Always	18 (23.7)	8 (25.8)	26 (19.1)	4 (10.8)	56 (20.0)
Trouble sleeping	Never	28 (36.4)	18 (58.1)	46 (33.6)	19 (51.4)	111 (39.4)
	Sometimes	33 (42.9)	9 (29.0)	68 (49.6)	17 (46.0)	127 (45.0)
	Always	16 (20.8)	4 (12.9)	23 (16.8)	1 (2.7)	44 (15.6)
Appetite changes	Never	47 (61.0)	13 (41.9)	81 (59.1)	31 (83.8)	172 (61.0)
	Sometimes	24 (31.2)	16 (51.6)	41 (29.9)	5 (13.5)	86 (30.5)
	Always	6 (7.8)	2 (6.5)	15 (11.0)	1 (2.7)	24 (8.5)

The overall mean depression and anxiety score was 8.5 (95% confidence interval: 8.1–8.9), and this ranged from 6.1 (5.2–6.9) among participants of “other” (double decker or bisexual) identity to 9.4 (8.7–10.1) among kothis. Among nirvan hijras, the mean depression and anxiety score was 8.5 (7.6–9.3) and it was 8.0 (6.9–9.1) among akwa hijras. Two-way ANCOVAs were conducted to examine the effects of gender identity and violence/rape on depression and anxiety score. There was a significant difference in the depression and anxiety score based on gender identity (*p*=0.003). There was no significant difference in the depression and anxiety score based on whether or not they had experienced physical violence in the previous 6 months (*p*=0.073) or whether or not they had been raped in the previous 12 months (*p*=0.566). There were no statistically significant interactions between gender identity and physical violence on depression and anxiety score [*F*(3, 255)=0.26, *p*=0.854] or gender identity and rape on depression and anxiety score [*F*(3, 250)=0.42, *p*=0.740].

## Discussion

Experiences of violence are common among gender-diverse individuals in Bangalore, South India. Nearly half of nirvan and akwa hijra study participants and a quarter of all kothi study participants had experienced physical violence in the previous 6 months, and this was associated with younger age and working as a laborer or transport worker or working in basti, sex work, chelas, or at a CBO. Those employed at CBOs are peer workers, and therefore likely have lived experience of working in basti, sex work, or chelas. Experiences of rape in the previous year were also common, especially among akwa hijras, and were associated with younger age and working in basti, sex work, as a chelas, or at a CBO.

These findings are consistent with other reports of the high incidence of violence experienced by hijras and kothis in South Asia.^43–45^ A 2012 survey of hijras in a region of Kolkata revealed that 17% had experienced sexual violence in the previous 3 months.^45^ Pervasive violence experienced by hijras from locals, clients, and even gurus was described by Ganju and Saggurti,^46^ and it was suggested that internalized stigma resulted in low-self efficacy to challenge abuse.^46^ A 2007 survey conducted in Abbottabad and Rawalpindi revealed similarly high levels of violence experienced by hijras and kothis in Pakistan in the previous year at the hands of neighbors (9% and 12%, respectively), police (32% and 18%, respectively), and clients (27% and 28%, respectively), and nearly one-third of hijras experiencing sexual violence at the hands of the police and clients.^44^ Another study of hijras and kothis in Pakistan also found that experiences of stigma, violence, and sexual abuse were common, and “among the most pressing problems facing feminized men in Pakistan today.”^47^

The findings that fewer participants with employment in the service industry, professionals, and those in trades had experienced physical violence in the previous 6 months and that more working in basti, sex work, as a chelas, or at a CBO had experienced rape in the previous year show how the exclusion of hijras and other gender-diverse individuals from education and formal labor translates into their vulnerability to violence.

Constant feelings of worry were common. Persistent trouble with sleeping and feelings of fatigue were reported by about one-fifth of nirvan hijras and kothis. Kothis reported constant feelings of sadness and loss of interest in daily activities more than any other group, and they had the highest calculated depression and anxiety scores.

In another survey among MSM, high levels of depression were also identified and were found to be associated with multiple types of stigma.^27^ Access to social supports and resilience were associated with lower depression.^27^ Feelings of shame, humiliation, internalized stigma, low self-worth were found to be common among hijra individuals in Maharashtra.^46^ Stigma, violence, and lack of family support were found to be linked to low self-esteem, depression, and suicidal ideation.^46^ Chakrapani et al.^28^ reported that two-thirds of the MSM and 91% of the transgender people in their study had at least one psychosocial health condition.^28^ They found that among MSM, victimization was significantly associated with higher odds of depression and the reverse was also found, whereby depression was significantly associated with higher odds of victimization.^28^ For both MSM and transgender people, the presence of depression was associated with sexual risk behavior,^28^ evidence which may be used to justify the inclusion of mental health services within HIV prevention programs. A study conducted among hijras in India provided further justification for this, revealing that moderate and high levels of victimization (including family nonacceptance, police harassment and assault, and discrimination) were associated with greater sexual risk related to inconsistent condom use, a high number of multiple sexual partners, and alcohol consumption before sex, placing them at greater risk of sexually transmitted infections, including HIV.^48^

The suicide rate among transgender individuals in India is said to be about 31%, with half attempting suicide before their 20th birthday.^49^ Violence, depression, and suicide are unacceptably high among gender-diverse individuals in India and require immediate action. There is a need to address the stigma, discrimination, and structural violence that produce conditions under which gender-diverse individuals in India are facing frequent physical and sexual violence, difficulty accessing health and social services, and the effects of poverty.

Facing similar levels of stigma and violence, members of the hijra community interviewed in Lahore and Karachi, Pakistan, raised the need for enforcement of human rights and suggested psychological counseling to deal with low self-esteem and develop skills to deal with the animosity directed toward them, as well as the establishment of networks of community members to mediate access to medical care and social services.^47^

Support from peers has been described as a common coping resource for hijras in India, offering protection from violence, financial assistance, and emotional support.^43^ Anecdotally, gurus have been described by some community members as a source of support, releasing hijras from police custody and providing financial support (personal communication with S. Dutta, January 2012). This may partially explain why hijra study participants tended to experience less sadness, less loss of interest in daily activities, and lower depression and anxiety scores compared to kothi study participants, which is consistent with Meyer's Minority Stress Theory.^27,31^ Another important source of support and moderator of violence is derived from the collective action of community networks.^46^ Local nongovernmental organizations (NGOs) may also offer advocacy with police, reducing violence; bail; and linkages to health services.^46^

Participation in community collectives has been demonstrated to improve the social capital and access to services for female sex workers in South India.^27,28,48–53^ Research conducted on the Pehchan program,^54^ which aims to strengthen community systems and provide access to health, legal, and social services for MSM, transgender people, and hijras across India, has similarly demonstrated the power of collectivization and gender-affirming approaches to improve demand and access to services.^12^ Similarly, Ganju and Saggurti write about the importance of community mobilization, particularly for transgender people in sex work in Maharashtra, India, to safeguard rights and reduce vulnerabilities.^46^

In the context of the 2014 Supreme Court of India decision, directing the government to officially recognize transgender people as a third gender and to develop programs specifically designed to meet their needs^10,55^ and the 2017 Mental Healthcare Act, the time to develop, fund, and deliver high-coverage mental health services, specifically designed for the needs of gender-diverse individuals, are now in India.

Changes are starting to take place. Karnataka's 2017 State Policy for Transgenders which aims to create awareness and address issues of discrimination and violence in educational institutions and reach out to family members to sensitize them about trans children was cleared in October 2017, in compliance with the Supreme Court order. Further action and frequent monitoring on the part of the Government of India are required to ensure that states meet their obligations to deliver mental health services to all of their residents, “without discrimination on the basis of gender, sex, sexual orientation, religion, culture, caste, social or political beliefs, class, disability or any other basis and provided in a manner that is acceptable to persons with mental illness and their families and caregivers.”^39^ In addition, action is required to reduce the endemic violence and harassment faced by gender-diverse individuals in India and provide support to those who experience violence and harassment.

Notably, the study participants in the “other” gender identity category, who identified as double-decker or bisexual, had fewer experiences of physical violence and rape than the other participants and fewer had mental health issues. These individuals are enrolled in the same HIV program as individuals with very different experiences and a much greater need for programs that address violence and mental health. It is important that programs have an understanding of the diversity of their clientele and tailor services to match their needs.

### Limitations

This study is limited by its cross-sectional design, and as a result, it is not possible to determine whether experiences of violence reported by participants caused their mental health issues. This study is subject to recall bias, as participants were asked to report experiences of violence from up to a year before their participation in the study. The results of this study are specific to gender-diverse individuals who were registered with an HIV prevention program in Bangalore, India, and are exploratory in nature. Subsequent qualitative and quantitative research is recommended to explore, in more depth, the occurrence of violence and of mental health concerns that have been illuminated through this research.

## Conclusions

Experiences of physical violence, rape, and poor mental health are common among gender-diverse individuals in Bangalore, Karnataka. The results of this study and others point to the need for specific actions which include the education and sensitization of medical and paramedical professionals, judiciary, police, counselors, and NGO staff around the lived experiences and needs of gender-diverse individuals to improve equitable and safe access to services; the establishment and strengthening of targeted helplines and mental health services; and the strengthening of the survivor support system for gender-diverse individuals who have been the target of violence.

## Abbreviations Used

ANCOVA: analysis of covariance
CBO: community-based organization
MSM: men who have sex with men
NGO: nongovernmental organization
SD: standard deviation

## Notes

^*^ The term “panthi” is used by kothis and hijras to refer to their male sexual partners.

^†^ “Double deckers” are individuals who act as both receptive and penetrative partner during anal and oral sexual intercourse between males.
